# Circadian genes *Per1* and *Per2* increase radiosensitivity of glioma *in vivo*

**DOI:** 10.18632/oncotarget.3179

**Published:** 2015-02-07

**Authors:** Niu Zhanfeng, Li Yanhui, Fei Zhou, Hao Shaocai, Li Guangxing, Xia Hechun

**Affiliations:** ^1^ Department of Neurosurgery, The General Hospital of Ningxia Medical University, Yinchuan, China; ^2^ Department of Neurosurgery, The Xijing Hospital of The Fourth Miltary Medical University, Xi'an, China; ^3^ Graduate School of Ningxia Medical University, Yinchuan, China; ^4^ Incubation Base of National Key Laboratory for Cerebrocranial Diseases, Ningxia Medical University, Yinchuan, China

**Keywords:** *Per1*, *Per2*, glioma, radiotherapeutic sensitivity

## Abstract

*Per1* and *Per2* play a key role in regulating the circadian rhythm in mammals. We report here that although both genes were expressed with a circadian rhythm in glioma and normal brain tissue in rats, their expression profiles differed in the two types of tissue. In addition, high expression of *Per1* and *Per2* in glioma tissue was associated with increased sensitivity to x-irradiation. No such sensitizing effect was observed in normal tissue. Our results suggest that *Per1* and *Per2* expression may increase the efficacy of radiotherapy against glioma by promoting apoptosis.

## INTRODUCTION

In organisms, healthy and cancerous cells alike have circadian clocks controlled by the same set of clock genes. You et al. reported characteristic chronobiological rhythms in a wide variety of tumors [1]. Studies in tumor-bearing mice suggest that optimizing the time when treatment is administered can reduce the toxicity of cytotoxic drugs, allow the use of higher doses, and improve treatment outcomes, including survival time [2]. Hori et al. [3] discovered that tumor tissue blood flow increased significantly at night. This suggests that the concentration of a drug reaching a tumor via the blood follows a circadian rhythm *in vivo*. Clinical studies have shown that using chronobiology to optimize the delivery time of the standard chemotherapy drug leads to the best outcomes in advanced colorectal cancer and pancreatic cancer [4, 5].

Circadian rhythms, and *Per* proteins in particular, may even influence cancer [6, 7]. Overexpression of Period1(*Per1*) and Period2(*Per2*) sensitizes human cancer cells to DNA damage-induced apoptosis, significantly increasing apoptosis in tumor cells [7–9]. In addition, *Per1* and *Per2* have been reported to be downregulated in several human cancers [10], and overexpression of either gene inhibits the growth of cancer cells [8, 11]. These studies show that high expression of *Per1* and *Per2* can promote tumor cell apoptosis. Therefore *Per* genes may act as tumor suppressors.

To test whether circadian expression of *Per1* and *Per2* may be involved in glioma growth, we analyzed expression patterns in both glioma and normal brain tissue. We found differences in the circadian expression, and we observed that in glioma tissue, *Per1* and *Per2* expression was positively correlated with apoptosis and negatively correlated with proliferation. These data further support the concept of chronobiology and provide an experimental basis for individualized treatment of glioma.

## RESULTS

### Rhythmic changes in *Per1* and *Per2* mRNA levels in normal and glioma tissues

We measured the expression of *Per1* and *Per2* mRNA over a period of 24 h in both normal and glioma tissue from rats. In normal tissues, the *Per1* mRNA level peaked at ZT8, after which it gradually decreased and had a nadir at ZT20–24 (Supplementary Figure S1A). And *Per2* mRNA expression levels began to rise immediately after ZT0, reached a peak at ZT12, and then gradually fell to a nadir at ZT24 (Supplementary Figure S1A). In contrast, in the glioma tissues, the *Per1* mRNA level peaked at ZT4, it was low at ZT12, then it hit a second peak around ZT16 and a second trough around ZT24 (Supplementary Figure S1B). And the level of *Per2* mRNA peaked at ZT24 (ZT0), fell to a low at ZT8, then rose to a second peak around ZT12, followed by a second trough around ZT20 (Supplementary Figure S1B).

### Effect of irradiation on *Per1* and *Per2* gene expression

**Figure 1:**
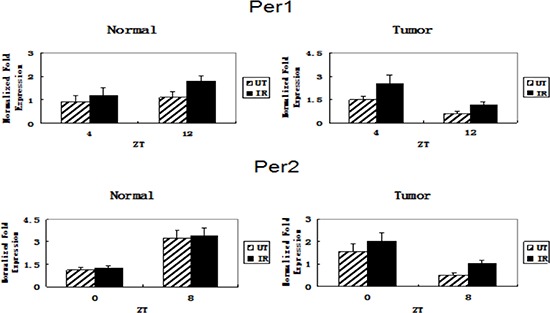
Changes in *Per1* and *Per2* mRNA expression in glioma and normal tissues after ionizing irradiation Glioma and normal tissues were treated with ionizing radiation (IR) or not (untreated, UT), then total RNA was extracted and used as template in real-time RT-PCR to measure *Per1* and *Per2* mRNA expression. The relative level of each mRNA was normalized to the corresponding level of β-actin mRNA. Data are the mean and standard deviation of three independent experiments.

We used real-time RT-PCR to measure *Per1* and *Per2* mRNA levels in glioma and normal tissues after treatment with ionizing radiation (15 Gy). In normal tissue, we compared *Per1* mRNA levels at ZT4, when *Per1* expression was high in glioma tissue. The levels did not differ significantly between the irradiated and untreated (control) groups at this time (*t* = –1.405, *p* > 0.05). However, when we compared the levels at ZT12, when *Per1* expression was low in glioma, the level in the irradiated group was significantly higher than in the control group (*t* = –5.135, *p* < 0.001, Figure 1). *Per2* mRNA levels did not differ significantly between the irradiated and untreated (control) groups at either ZT0 (*t* = –1.742, *p* > 0.1) or ZT8 (*t* = –0.642, *p* > 0.5) (Figure 1), when *Per2* expression in glioma tissue was high or low, respectively. In glioma tissue, the levels of *Per1* and *Per2* mRNA were significantly higher in the irradiated group than in the control group when *Per1* and *Per2* expression was elevated, i.e. at ZT4 for *Per1* (*t* = –4.464, *p* < 0.001) and ZT0 for *Per2* (*t* = –2.407, *p* < 0.05). Similar results were observed when *Per1* and *Per2* expression was low, i.e. at ZT12 for *Per1* (*t* = –5.276, *p* < 0.001) and ZT8 for *Per2* (*t* = –6.599, *p* < 0.001) (Figure 1).

### Negative correlation between cell proliferation and *Per1* and *Per2* mRNA levels in glioma tissue

**Figure 2:**
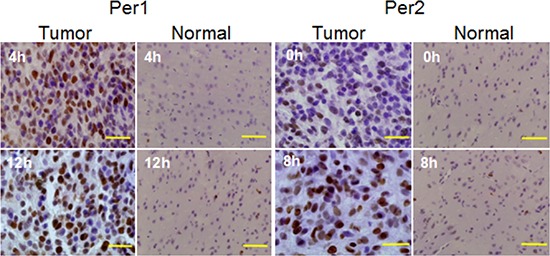
PCNA-based detection of proliferation in glioma and normal tissue subjected to x-irradiation at times (ZTs) when *Per1* and *Per2* mRNA levels were high and low Scale bar in tumor column, 20 μm; scale bar in normal column, 50 μm.

We measured cell proliferation in glioma and normal tissues at ZTs when *Per1* and *Per2* mRNA levels were high and low, following irradiation with a single dose of x-radiation (15 Gy). In glioma tissues, *Per1* expression showed a period of 12 h. The proportion of proliferating cells at ZT4 (44.8%), when *Per1* expression was at its peak, was not significantly different from the proportion at ZT12 (46.7%, *p* > 0.05), when *Per1* expression was at a nadir. Similar results were obtained in normal tissue, where the proportion of proliferating cells was not significantly different between ZT4 (0.002%) and ZT12 (0.02%, *p* > 0.05) (Figure 2).

Proliferation in glioma tissue correlated with the level of *Per2* expression (Figure 2). The proportion of proliferating cells at ZT0 (27.3%), when *Per2* expression was maximal, was significantly lower than at ZT8 (63.9%, *p* < 0.001), when *Per2* expression was at a nadir. In contrast, in normal tissue, the proportion of proliferating cells was not significantly different between ZT0 (0.016%) and ZT8 (0.027%, *p* > 0.05).

These results show an association between endogenous *Per* expression and proliferation after x-irradiation.

### Positive correlation between apoptosis and *Per1* and *Per2* mRNA levels in glioma tissue

**Figure 3:**
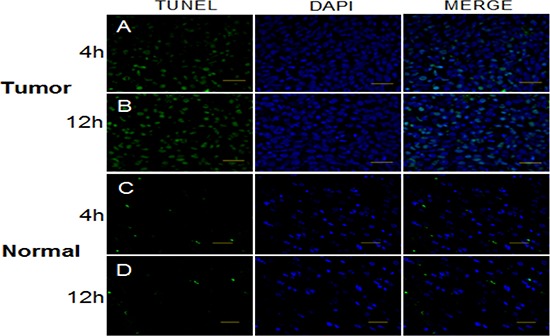
TUNEL assay to detect apoptosis in glioma and normal tissue at times of high and low expression of *Per1* following a single dose of x-radiation (15 Gy) Bar, 50 μm.

We detected apoptosis in glioma and normal tissue after irradiating animals at times when *Per1* and *Per2* mRNA levels were high and low. In glioma tissue, *Per1* expression showed a period of approximately 12 h (Figure 3A, 3B). The proportion of apoptotic cells at ZT4 (28.1%, *p* > 0.05), when *Per1* expression was at its peak, was not significantly different than at ZT12 (31.7%, *p* > 0.05), when *Per1* expression was at a nadir. Similarly, in normal tissue (Figure 3C, 3D), the proportion of apoptotic cells was not significantly different between ZT4 (7.0%, *p* > 0.05) and ZT12 (6.1%, *p* > 0.05).

**Figure 4:**
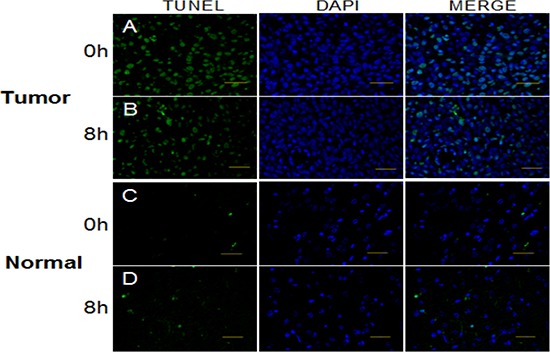
TUNEL assay to detect apoptosis in the glioma and normal tissues at times of high and low expression of *Per2* after a single dose of x-radiation (15 Gy) Bar, 50 μm.

Apoptosis in glioma tissue correlated with the level of *Per2* expression (Figure 4A, 4B). The proportion of apoptotic cells at ZT0 (35.0%, *p* < 0.001), when *Per2* expression was maximal, was significantly higher than at ZT8 (21.5%, *p* < 0.001), when *Per2* expression was at a nadir. In contrast, in normal tissue (Figure 4C, 4D), the proportion of apoptotic cells was not significantly different between ZT4 (7.7%, *p* > 0.05) and ZT12 (9.2%, *p* > 0.05).

These results show an association between endogenous *Per* expression and apoptosis level after x-irradiation.

## DISCUSSION

Our former study has showed that *Per1* and *Per2* expression abnormalities can be associated with the occurrence of glioma [15]. In this research, we demonstrated that the expression of the clock oscillatory genes *Per1* and *Per2* shows a circadian rhythm in both glioma and normal brain tissue. However, the profile of the oscillation differs between the two types of tissue. The expression of *Per1* and *Per2* in glioma tissue shows a period of approximately 12 h, but in normal tissue it shows a period of approximately 24 h. The oscillation in *Per* expression in glioma tissue may indicate that it can generate circadian rhythms, and this circadian oscillation may be regulated by SCN. Cultured astrocytes, which also show circadian oscillation of clock gene activity [16], sustained these rhythms when co-cultured with explants of adult SCN, but not with cortical explants [17].

We x-irradiated normal and glioma tissue at times when *Per1* and *Per2* mRNA levels were high and low in gliomas. We observed that *Per1* and *Per2* mRNA levels in irradiated glioma tissue were significantly higher than in untreated glioma. In normal tissue, however, the expression of *Per1* and *Per2* was similar in irradiated and control tissue, except at ZT12, when *Per1* expression was significantly higher in irradiated tissue. Previous studies showed that irradiation upregulates transcription of a number of murine clock genes, including *Per1* and *Per2* [7]. This suggests that x-irradiation can increase the expression of *Per1* and *Per2* in glioma tissue.

To determine whether changes in *Per1* and *Per2* expression may affect the radiation sensitivity of glioma, we measured proliferation and apoptosis of glioma cells when rats were x-irradiated at times when *Per1* and *Per2* mRNA levels were high and low. Proliferation and apoptosis of glioma cells showed no significant difference when *Per1* expression was high (ZT4) or low (ZT12). In contrast, proliferation was significantly lower and apoptosis significantly higher in glioma cells expressing high levels of *Per2* (ZT0) than in cells expressing low levels (ZT8). This suggests that the low proliferation and high apoptosis associated with low *Per1* expression in glioma may result from the coincidence at ZT12 of high *Per2* expression and low *Per1* expression. According to this hypothesis, the high proliferation and low apoptosis normally associated with low *Per1* expression is masked at ZT12 by the low proliferation and high apoptosis associated with high *Per2* expression.

DNA damage from agents that cause double-strand breaks (DSBs), such as ionizing irradiation, is one of the most harmful DNA lesions. When DSBs occur, cells either activate their replication checkpoints to delay S phase progression and G2/M transition, or when faced with irreparable damage, they activate their apoptotic machinery [18, 19]. As the core circadian genes, *Per1* and *Per2* not only maintain the circadian rhythm of cells, they also sustain the normal cell cycle by regulating the expression of cell cycle-related genes such as *p53, c-Myc* and *cyclinB1* [7, 19, 20]. Fu et al. [7] reported that when wild-type mice and mice carrying two defective copies of the mouse *Per2* gene (*mPer2^m/m^*) were treated with x-radiation, the ratio of apoptotic thymocytes was about 2-fold higher in wild-type thymus; in addition, the level of *p53* protein in mice was lower than in wild-type mice. The levels of *p53* mRNA and protein were higher in Lewis lung carcinoma (LLC) cells than in vector control cells. This suggests that overexpression of *mPer2* induces *p53* expression, which then suppresses tumor growth [11, 21]. This is consistent with studies in *Per2-*overexpressing leukemia cells [9]. When mammalian cells contain damaged DNA, the *p53* tumor suppressor and the *Rb* family of transcriptional repressors work together to downregulate a large number of genes that encode proteins required for the G2/M transition. Elimination of these essential cell cycle proteins helps to keep the cells arrested in G2 [22]. Another study showed that *p53* is a *Cdk1-CyclinB1* regulator that may inhibit *Cdk1-CyclinB1* transcription, leading to cell cycle arrest at G2/M [23]. Moreover, circadian regulation of *CyclinB1* appears to be indirect and to occur via a *p53*-dependent mechanism, with *p53* helping to block entry into mitosis and strengthen G2 arrest [24]. These findings suggest that the high expression of *Per1* and *Per2* observed after irradiation in our study upregulated *p53* expression, which arrested the cells at G2/M. In addition, the high expression of *Per1* and *Per2* sensitized the glioma cells to x-irradiation, promoting apoptosis as a result.

Our findings extend previous studies showing that overexpression of *Per1* and *Per2* promotes apoptosis by altering the expression of apoptosis-related genes [7, 19, 20], including in prostate cancer cells [25]. Overexpression of *Per1* and *Per2* sensitized human cancer cells to DNA damage-induced apoptosis, while inhibiting expression of these genes in similarly treated cells reduced apoptosis [19, 20]. Transfecting human cancer cell lines with *Per1* and *Per2* expression plasmids led to cessation of growth [8, 9, 11].

The *c-Myc* oncoprotein is a transcription factor that promotes cell growth and proliferation, as well as apoptosis under certain conditions. Activation of the *c-Myc* oncoprotein is a frequent event in human cancer and has been associated with almost every aspect of tumorigenesis [26, 27]. Abnormal expression of *c-Myc* has been suggested as the cause of malignant growth in mice with disrupted circadian coordination [28]. In addition, deregulated expression of *c-Myc* has been suggested as a key factor leading to tumor development in *Per2* mutant mice [7]. When radiation-induced DSBs were introduced in LLC cells overexpressing *mPer2*, *c-Myc* mRNA and protein levels decreased below the levels in empty-vector control cells. The overexpression of *Per2* induces *Bmal1* expression and then increases intracellular levels of *Bmal1/Npas2* or *Bmal1/Clock* proteins, in addition to repressing *c-Myc* [11]. Furthermore, the p53 protein binds to the *c-Myc* promoter *in vivo* and represses it through a mechanism that involves histone deacetylation [29]. Thus, overexpression of *Per2* may promote apoptosis in glioma tissue by downregulating *c-Myc* and upregulating *p53*.

Conversely, previous research has shown that irradiation of *Per1-*overexpressing cells led to considerable upregullation of *c-Myc*, and irradiation induced *p21 ^Waf1/Cip1^* accumulation in cells transfected with empty vector, whereas this response was markedly attenuated in HCT116 cells overexpressing *Per1*.^20^ Thus, *Per1* may sensitize cells to DNA damage-induced apoptosis by elevating *c-Myc* and suppressing *p21^Waf1/Cip1^* [8, 30]. This suggests that *Per1* and *Per2* promote apoptosis through different mechanisms.

Our results suggest that *Per1* and *Per2* act as tumor suppressor genes in gliomas and that their high expression can induce cell cycle arrest and increase tumor sensitivity to x-rays through a p53-dependent mechanism. This is consistent with previous studies showing that *Per1* is a tumor suppressor gene in non-small lung cell cancer [31] and prostate cancer [25]. The radiosensitivity of cells appears to depend on where they are in the cell cycle, with G2/M phase the most sensitive period [32]. Peak expression of *Per1* and *Per2* during the day may have effects similar to those of other agents that arrest cells in G2/M and that are known to be effective radiosensitizing agents [33, 34], such as celecoxib, gefitinib, and tachpyridine [35]. Khapre RV et al. [36] also discussed the circadian rhythm which is driven by mTOR and is different from other diurnal clocks. Importantly, fasting and calorie restriction inhibits mTOR [37], and as a result fasting may improve cancer therapy [38]. Also, aging is associated with cancer and poor response to therapy. This may be explained in part by alterations of circadian rhythm [39–43]. Future research should focus on the detailed mechanisms by which *Per1* and *Per2* regulate the expression of genes related to cell proliferation and apoptosis in gliomas. Further studies should also test the potential survival benefit of chronotherapy in gliomas and develop optimal scheduling guidelines for radiation therapy.

## MATERIALS AND METHODS

### Cell culture

C6 cells were cultured in Dulbecco's Modified Eagle's Medium (DMEM, Hyclone, catalog no. SH30022.01B) supplemented with 10% fetal bovine serum (FBS, Hyclone, catalog no. SV30087.01) at 37°C with 5% CO_2_.

### Animal experiments

These studies were conducted in accordance with the animal care guidelines instituted by the Animal Studies Committee of Ningxia Medical University.

Sprague–Dawley male rats (*n* = 160, 120–150 g) were obtained from Ningxia Medical University Experimental Animal Center. The rats were housed under a standard light/dark cycle of 12 h:12 h at 24 ± 1°C. In this study, times are reported as Zeitgeber time (ZT), or hours after light onset. Thus, ZT 0 indicates when lights were turned on and ZT 12 when lights were turned off. The animals were adapted to the 12 h:12 h light/dark cycle for 3 weeks before the experiments. The glioma rat model was established according to Watanabe's method [12]. Animals were anesthetized for irradiation and perfusion procedures; anesthesia consisted of an i.p. injection of sodium pentobarbital (50 mg/kg).

To examine the expression of *Per1* and *Per2* genes in rat glioma and normal tissue over 24 hours, we first examined mRNA expression during this period. Ten animals were killed by anesthesia (100 mg/kg) every 4 h, i.e. at ZT4, ZT8, ZT12, ZT16, ZT20, and ZT24 (ZT0). Tumor tissue and contralateral normal tissue were rapidly removed and frozen in liquid nitrogen. Tissue was then processed for quantification of *Per1* and *Per2* mRNA by real-time RT-PCR.

In order to determine the effects of irradiation on glioma and normal tissue when *Per1* and *Per2* mRNA levels were high and low, tumor-bearing rats (*n* = 60) were randomized into six groups, and 10 rats were administered a single 15-Gy dose of x-rays (Varian 2100C/D, USA) at each of the following time points: ZT4, ZT8, ZT12, ZT16, ZT20, ZT24. At 6 h after irradiation [7, 13], rats were killed by anesthesia (100 mg/kg) and perfused with 4% paraformaldehyde. The brains were fixed overnight in 10% phosphate-buffered formalin (PBF), rinsed in 70% ethanol, and paraffin-embedded.

In order to observe the expression of *Per1* and *Per2* mRNA in glioma and normal tissue after irradiation, tumor-bearing rats (*n* = 40) were randomized into four groups and subjected to 15 Gy of radiation at times when *Per1* and *Per2* mRNA levels were high and low. The tumor tissue and contralateral normal tissue were rapidly removed and frozen in liquid nitrogen. This tissue was used for quantification of *Per1* and *Per2* mRNA by real-time RT-PCR.

### Real-time RT-PCR

Total RNA from glioma and normal tissues was extracted using Trizol reagent (Invitrogen, catalog no. 15596026). RNA (2 μg) was reverse-transcribed into cDNA using the cDNA synthesis kit (Invitrogen, catalog no. 11917020) following the manufacturer's instructions. PCR primers for rat *Per* (*rPer*) used in this study were as follows: *rPer1* sense, 5-GGTCACGGAGTCATCCAATCA-3; *rPer1* antisense, 5-ATCCCGAACCAGACCCAGAG-3; *rPer2* sense, 5-GTGAAAGTGAGGAGAAAGGCAACC-3; *rPer2* antisense, 5-CACCTCTTCCGAGCACCGTC TA-3; *β-actin* sense, 5-CCCATCTATGAGGGTTACGC-3; *β-actin* antisense, 5-TTTAATGTCACGCACGATTTC-3. In each set of six samples run in parallel, one sample was used to run a calibration reaction. Relative expression of *Per1* and *Per2* mRNA levels was determined using the relative quantification method and 2^−ΔΔCt^ analysis [14].

### Cell proliferation analysis

We measured cell proliferation in glioma and normal tissue by determining levels of proliferating cell nuclear antigen (PCNA). Sections (4 μm) of paraffin-embedded tissue were labelled with mouse monoclonal antibodies against PCNA (1:100 dilution, Boster, catalog no. BM0104, Wuhan, China). After blocking the tissue with goat serum to reduce any non-specific binding to the conjugated second antibody, the samples were incubated overnight at 4°C with primary antibodies. After three washes with phosphate-buffered saline (PBS, pH 7.2), a secondary antibody kit (Bioss, catalog no. SP-0024, Beijing, China) was used according to the manufacturer's instructions. Immunostaining was performed with diaminobenzidine (DAB). Slides were counterstained with hematoxylin for 3 min, dehydrated through a graded series of ethanol solutions, placed into xylene for 5 min, and coverslipped. As a negative control, primary antibody was replaced with PBS. Slides were examined by light microscopy (Olympus BX-61).

### TUNEL assay

To detect apoptotic cells in tumor and normal tissue, sections were analyzed by terminal deoxynucleotidyl transferase-mediated deoxyuridine triphosphate-biotin nick end-labeling (TUNEL, Roche, 11684817910) according to the manufacturer's instructions. After deparaffinization, sections were treated with proteinase K (20 mg/ml in 10 mM Tris/HCl, pH 7.4–8.0) for 30 min at 37°C. Then the sections were incubated with TUNEL reaction mixture for 60 min at 37°C in a humidified atmosphere in the dark. The slides were rinsed 3 times with PBS, coverslipped using VectaSHIELD mounting medium containing DAPI (Vector Laboratories, catalog no. H-1200), and analyzed by laser scanning confocal microscope (Olympus FV1000 Viewer). Excitation wavelengths were in the range of 450–500 nm and detection wavelengths in the range of 515–565 nm (green).

### Statistical analysis

Real-time PCR results are reported as mean ± standard deviation and were analyzed statistically using the ANOVA test and Student's *t*-test. The Pearson chi-square test was used to compare apoptosis and proliferation results between groups. *P* values of less than 0.05 were considered statistically significant.

## SUPPLEMENTARY FIGURE


