# Supplementary material for: Circadian genes Per1 and Per2 increase radiosensitivity of glioma in vivo
Source: Oncotarget. 2015 Feb 7;6(12):9951–8. doi: 10.18632/oncotarget.3179 (PMC4496409; doi:10.18632/oncotarget.3179)
Supplement: Supplementary file 1 [file oncotarget-06-9951-s001.pdf]

SUPPLEMENTARY FIGURE

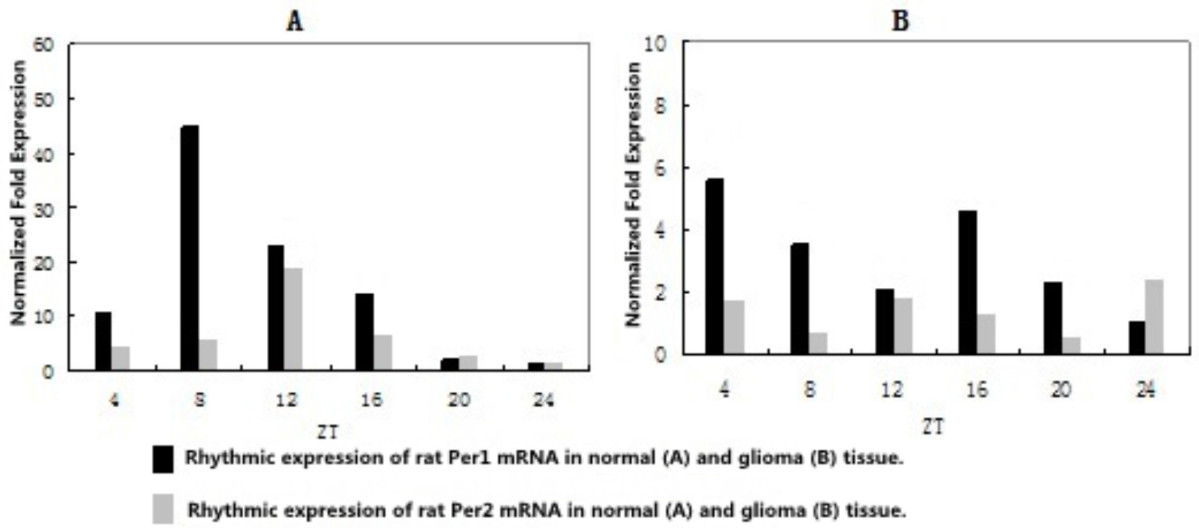

Supplementary Figure S1: Rhythmic expression of rat *Per1* and *Per2* mRNA in normal (A) and glioma (B) tissue. Total RNA was extracted from the SCN, and real-time RT-PCR was used to determine *Per1* and *Per2* mRNA levels. Relative expression of each mRNA was normalized to the corresponding level of β-actin mRNA. Data are the mean and standard deviation of three independent experiments.
